# Radiographic Imaging for the Diagnosis and Treatment of Patients with Skeletal Class III Malocclusion

**DOI:** 10.3390/diagnostics14050544

**Published:** 2024-03-04

**Authors:** Zhuoying Li, Kuo Feng Hung, Qi Yong H. Ai, Min Gu, Yu-xiong Su, Zhiyi Shan

**Affiliations:** 1 Division of Paediatric Dentistry and Orthodontics, Faculty of Dentistry, The University of Hong Kong, Hong Kong SAR, China; lizhuoying@connect.hku.hk (Z.L.); drgumin@hku.hk (M.G.); 2 Applied Oral Sciences & Community Dental Care, Faculty of Dentistry, The University of Hong Kong, Hong Kong SAR, China; hungkfg@hku.hk; 3 Department of Health Technology and Informatics, The Hong Kong Polytechnic University, Hung Hom, Kowloon, Hong Kong SAR, China; hemis.ai@polyu.edu.hk; 4 Division of Oral and Maxillofacial Surgery, Faculty of Dentistry, The University of Hong Kong, Hong Kong SAR, China; richsu@hku.hk

**Keywords:** artificial intelligence, Class III malocclusion, diagnosis and treatment, radiographic imaging

## Abstract

Skeletal Class III malocclusion is one type of dentofacial deformity that significantly affects patients’ facial aesthetics and oral health. The orthodontic treatment of skeletal Class III malocclusion presents challenges due to uncertainties surrounding mandibular growth patterns and treatment outcomes. In recent years, disease-specific radiographic features have garnered interest from researchers in various fields including orthodontics, for their exceptional performance in enhancing diagnostic precision and treatment effect predictability. The aim of this narrative review is to provide an overview of the valuable radiographic features in the diagnosis and management of skeletal Class III malocclusion. Based on the existing literature, a series of analyses on lateral cephalograms have been concluded to identify the significant variables related to facial type classification, growth prediction, and decision-making for tooth extractions and orthognathic surgery in patients with skeletal Class III malocclusion. Furthermore, we summarize the parameters regarding the inter-maxillary relationship, as well as different anatomical structures including the maxilla, mandible, craniofacial base, and soft tissues from conventional and machine learning statistical models. Several distinct radiographic features for Class III malocclusion have also been preliminarily observed using cone beam computed tomography (CBCT) and magnetic resonance imaging (MRI).

## 1. Introduction

As one type of malocclusion classified by Edward H. Angle, Class III malocclusion is a sagittal positional discrepancy that is characterized by a mesial molar relationship [1]. It was reported to affect over 7% of the global population and the prevalence is even higher in Southeast Asians, ranging from 12.58% to 26.67% [2]. Notably, the majority of Class III malocclusion cases display skeletal discrepancies to varying degrees. Skeletal Class III malocclusion patients exhibit a concave facial type due to maxillary retrusion and/or mandibular prognathism, suffering from the negative impact on their oral health, facial aesthetics, psychosocial well-being, and oral health-related quality of life [3,4,5]. When addressing skeletal Class III malocclusion, various treatment strategies are available, including early orthopedic treatment during growth, camouflage orthodontic treatment, and orthognathic surgery after growth completion [6,7]. However, choosing the appropriate treatment and determining the intervention timing can be challenging, especially for less-experienced orthodontists. The difficulties in treating skeletal Class III malocclusion stem from variations in mandibular growth patterns, diverse treatment options, a high risk of relapse, and irreversible changes following orthodontic extractions [8]. Generally, orthodontists make subjective decisions on the treatment of skeletal Class III malocclusion, relying on their prior training or experience, which may lead to a potential impact on the accuracy of treatment selection.

Radiographic imaging, such as cephalograms, cone beam computed tomography (CBCT) and magnetic resonance imaging (MRI), is a non-invasive examination that provides visual representations of dentoskeletal characteristics in orthodontics. There exist numerous radiographic features that could potentially aid in decision-making for skeletal Class III cases in clinical practice. Previous studies have focused on developing various analytic models to generalize these disease-specific radiographic features. Early in the 1970s, Schulhof et al. built a simple formula based on the measurements in lateral cephalograms, for the first time, to predict the facial growth of skeletal Class III malocclusion patients [9]. Later, significant indicators for the diagnosis and treatment of Class III malocclusion were identified by conventional statistical models, including cluster analysis, discriminant analysis, and regression analysis [10,11,12]. In the 1980s, Stensland et al. identified certain predictors for the relapse of Class III malocclusion in children after combined retractor and chin-cup therapy using discriminant analysis [11]. Nevertheless, although plenty of indicators for the diagnosis and treatment of Class III malocclusion have been investigated through conventional models, it is still hard to find them with consensus and satisfactory accuracy [13].

In the past few years, artificial intelligence (AI) technology, including machine learning (ML) algorithms, has witnessed a rapid advancement in identifying valuable radiographic features based on the measurement input. Thanks to its capacity to process enormous amounts of data through high-dimensional analytical methods, AI technology has been dramatically applied in orthodontic diagnosis, such as cephalometric analysis and skeletal-maturation-stage determination, treatment planning, such as treatment outcome prediction, as well as clinical practice, such as remote care [14]. Specifically, ML has been used for the diagnosis and treatment of Class III malocclusion to improve prediction accuracy, which is expected to aid in the diagnosis and treatment planning of Class III malocclusion cases, especially for non-specialists [8]. In 2009, Kim et al. compared the prediction accuracy between the ML algorithm and traditional discriminant analysis for the prediction of Class III malocclusion treatment outcomes among children, where the authors found that the ML analysis might be an effective alternative to the conventional model for prognosis prediction [13]. With increasing attention given to the value of medical images themselves, the requirement for quantitative imaging analysis gave rise to radiomics in 2012 [15]. Radiomics is an approach enabling the extraction of a large number of quantitative features in medical images and providing a detailed characterization of the underlying tissue properties [15]. It has been explored in the automatic diagnosis and prognosis prediction of maxillofacial diseases, particularly in detecting head and neck tumors [16,17].

Based on various analytic models, numerous radiographic features have been identified for skeletal Class III malocclusion. Staying updated on the latest findings regarding these radiographic features is essential for clinicians to make informed decisions in diagnosing and treating patients, and for researchers working on developing robust prediction models to enhance the understanding and management of skeletal Class III malocclusion. In a previous study, Piotr Fudalej et al. conducted a review of significant predictors for early orthodontic or orthopedic treatment outcomes in children with Class III malocclusion [18]. However, this review did not address radiographic parameters for all patients with skeletal Class III malocclusion, especially those requiring orthognathic surgery. Additionally, the analysis using advanced technologies such as ML has not been examined and updated. Therefore, the aim of this narrative review is to provide a comprehensive summary of radiographic features associated with patients with skeletal Class III malocclusion in the application of facial type classification, growth prediction, and decision-making for tooth extractions and orthognathic surgery. This review outlines radiographic features from both conventional statistical models and ML algorithms across multiple domains, encompassing inter-maxillary relationships and different anatomical structures. Furthermore, the review discusses the potential future application of radiographic features in diagnosing and treating skeletal Class III malocclusion.

## 2. Radiographic Features for Inter-Maxillary Relationship in the Diagnosis and Treatment of Skeletal Class III Malocclusion

**Figure 1:**
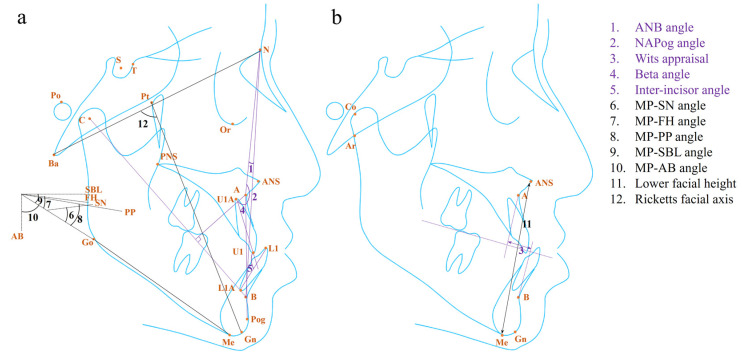
The (**a**) angular, (**b**) linear, and proportional measurements related to the sagittal (purple) and vertical (black) inter-maxillary relationship for diagnosis and treatment of skeletal Class III malocclusion in the existing literature. For the proportional values, only the involved landmark was labeled. The detailed information for the landmark, plane, and measurement is listed in Table S1.

Since the jaw’s development follows a specific spatiotemporal pattern, peaking in the transverse, sagittal, and vertical dimensions in sequence, we show the parameters in radiographic images involving inter-maxillary relationship by dimensions (Table 1 and Figure 1) [19].

### 2.1. Radiographic Features for Sagittal Inter-Maxillary Relationship

Patients with skeletal Class III malocclusion are mainly characterized by the concave facial type in the sagittal dimension. The parameters identified in this dimension, including the ANB angle, Wits appraisal, beta angle, jaw length ratio, and inter-incisor angle, hold great significance in the precise diagnosis of and treatment plan-making for Class III malocclusion.

As one of the most widely used means for evaluating the antero–posterior relationship of the jaws, the ANB angle, introduced by Riedel in 1952, was sometimes used to define the skeletal Class III malocclusion (Figure 1a) [40]. It was reported as one of the best predictors of relapse after treatment for Class III malocclusion in children, as well as facial type classification among children and adults [20,21,24]. In addition, it was one of the components that explained the variance of the mandibular prognathic subtype and the borderline Class III subtype [12]. Instead of the ANB angle, some researchers identified the NAPog angle as the predictor for the relapse of Class III malocclusion in children, where there was a higher risk of relapse in patients with a larger NAPog angle (Figure 1a) [22]. It suggested that the ANB angle might not be a perfect parameter without considering the chin protrusion.

Without the disturbance by the displacement of nasion, the Wits appraisal could be the most decisive parameter in the sagittal dimension for Class III malocclusion as it was identified most often from various studies (Figure 1b) [8,12,27,29,30,32,33,34,35]. In the past, Chi et al. recognized the Wits appraisal as one of the principal components that explained the variance of the borderline Class III subtype [12]. In those cases, the Wits appraisal may work together with the ANB angle as they belong to different reference systems and need to be considered at the same time. For growing patients with skeletal Class III malocclusion, a smaller Wits appraisal was later identified as a negative predictor for the long-term success of treatment for Class III malocclusion, including face mask, chin-cup, headgear, and fixed appliance therapy [24,25,27,28]. However, the critical value of the Wits appraisal for orthopedic treatment outcome prediction remained unclear, due to the limitation of statistical models. Most conventional models proposed in the literature provided total critical scores calculated from the statistical formulas, rather than individual critical scores for each significant variable, considering that it was the collective effect of the variables that helped with diagnosis or prediction. In addition, the Wits appraisal was of value in terms of decision-making for tooth extraction and surgery. Recently, a ML model revealed a positive relationship between the Wits appraisal and the likelihood of the need for orthodontic extractions in patients with different types of malocclusions [29]. Notably, the result needs to be interpreted with caution as it could be affected by the study sample which included patients with Class Ⅱ malocclusion. For patients who have completed the growth stage, the Wits appraisal is a useful parameter for determining the need for orthognathic surgery through the discriminant analysis and *t*-test [30,31,32,33]. Among them, Sara et al. proposed that the cut-off value of the Wits appraisal is −5.8 mm, which should be taken into consideration together with the Holdaway angle when making a treatment plan [33]. Moreover, various ML models built in the previous studies identified the Wits appraisal as a critical parameter contributing to the determination of the need for Class III surgery, further verifying its significance [8,34,35].

Introduced by Chong et al. in 2004, the beta angle was a new measurement for assessing the skeletal discrepancy that combined three skeletal landmarks—point A, point B, and the center of the condyle (C) (Figure 1a) [41]. It was reported as the top contributor for treatment plan prediction, including the orthodontic extraction pattern in skeletal Class Ⅰ patients, as well as growth modulation, camouflage orthodontic treatment, and orthognathic surgery in skeletal Class Ⅱ and Class III patients of different ages based on ML algorithms [34]. However, the information on how the beta angle contributed to the orthodontic treatment plan prediction was not provided in this study.

Besides the linear and angular parameters mentioned above, the proportion of the length of the maxilla and mandible was another predictor in the sagittal dimension. It was one of the contributors that explained the variance of the Class III subtype with maxillary deficiency and a long face, while the authors did not show specific measurements to assess the dimension [12]. In terms of treatment stability prediction, a larger ratio of Co-A to Co-Gn can help to predict better stability in the occlusal correction with a threshold of 0.74 in adolescents receiving conventional orthodontic treatment by means of removable and fixed appliances (Figure 1b) [23]. The comparative parameter related to the difference between individual jaw lengths and normal values also played a role [23]. In addition, a dynamic ratio called the Growth Treatment Response Vector (GTRV) that describes the change in the sagittal position of points A and B during a period can help predict the stability of treatment outcomes in children, where patients with a GTRV ratio below 0.38 should be warned of potential worsening discrepancies [26]. As for treatment plan-making, the smaller proportion of the maxillary and mandibular length is an indicator of surgery need in adult patients through the discriminant analysis [30,31]. Further, the ratio of A-Ar to Gn-Ar was identified as the predictor for orthognathic surgical need through the ML model (Figure 1b) [8]. However, the ratio might be affected by the direction and the spatiotemporal difference in jaw growth.

In addition to the skeletal variables, the dental parameters, such as the inter-incisor angle, explained the variance of the same severe Class III facial subtype, similar to the jaw length ratio, since they depend on the compensatory inclination of the incisors in serious skeletal Class III malocclusion cases (Figure 1a) [12]. According to the discriminant analysis, the inter-incisor angle was a predictor for relapse in Class III children after receiving both retractor and chin-cup therapy, where those with a larger angle tended to relapse after the treatment [11]. For adult patients, a larger angle meant a greater likelihood of orthognathic surgery need [32]. However, no threshold value was provided in the above studies.

### 2.2. Radiographic Features for Vertical Inter-Maxillary Relationship

During the later stage of the growth peak, the mandibular growth witnesses a profound growth in the vertical dimension, especially in some patients with severe Class III malocclusion. Here, we summarize the parameters in the vertical dimension, including the measurements based on the mandibular plane, facial height, and Ricketts facial axis.

The vertical measurement is indivisible with a core plane, namely, the mandibular plane (MP). The angles which are formed by MP and the SN plane or the Frankfort horizontal (FH) plane have been identified as the parameters for the classification of a vertical facial type in Class III patients using the conventional method and the ML model (Figure 1a) [12,21]. In terms of Class III malocclusion treatment, the research found a larger angle formed by MP and the palatal plane (PP) or the stable basicranial line (SBL) was a negative predictor for the relapse of Class III malocclusion among children receiving early functional treatment, and for the orthognathic surgery need among adults, which could eliminate the interference from the variation of the SN and FH plane (Figure 1a) [8,36,37]. In other studies, it was the MP-AB angle that helped predict the treatment outcome in adolescents under orthopedic treatment, where a smaller MP-AB angle hinted at a greater likelihood of Class III malocclusion relapse (Figure 1a) [13,27,38]. This could be explained by the advantage of the MP-AB angle in reflecting the discrepancy both in the sagittal and vertical dimensions without being affected by any reference planes.

The facial height in the upper and lower parts, anterior and posterior parts can directly describe diverse vertical facial types. Bui et al. identified facial height as the parameter that explained the variance of the vertical facial subtype of skeletal Class III patients [12]. Besides, Yoshida et al. concluded that a longer distance of ANS-Me, which described a lower facial height (LFH), could help predict the tendency of relapse among children under maxillary protraction and chin-cup therapy (Figure 1b) [39]. However, it should be noted that the facial height in different parts needs to be considered together as its coordination affects facial aesthetics.

The Ricketts facial axis was defined as the NBa-PtGn angle (Figure 1a) [42]. It was one of the predictors supporting decision-making for orthodontic extraction in patients with different kinds of malocclusion according to the ML model [29]. The study found patients with a smaller angle were prone to receive orthodontic extraction treatment [29]. It should be noted that the study sample included patients with different types of malocclusion, which may influence the precise predictability of this angle for determining the orthodontic extraction pattern, specifically in Class III malocclusion patients.

## 3. Radiographic Features of Different Anatomical Structures in the Diagnosis and Treatment of Skeletal Class III Malocclusion

**Figure 2:**
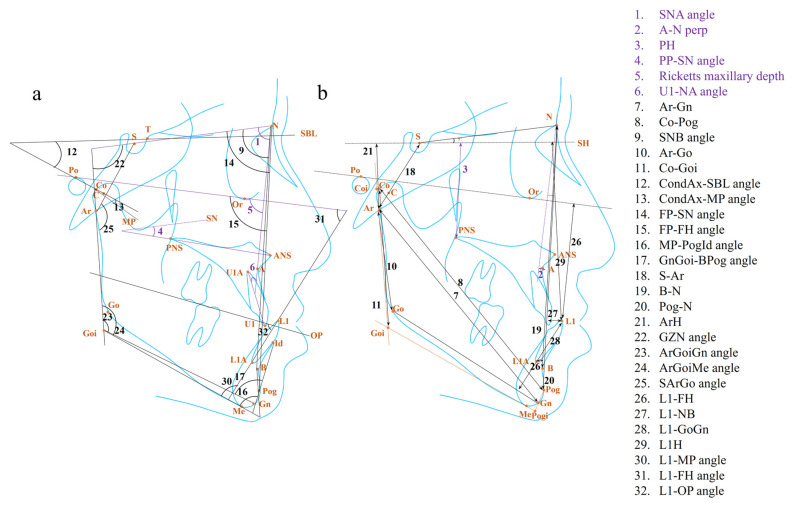
The (**a**) angular, (**b**) linear, and proportional measurements in the maxillary (purple) and mandibular (black) dimension for diagnosis and treatment of skeletal Class III malocclusion in the existing literature. For the proportional values, only the involved landmark was labeled. The detailed information for the landmark, plane, and measurement is listed in Table S1.

**Figure 3:**
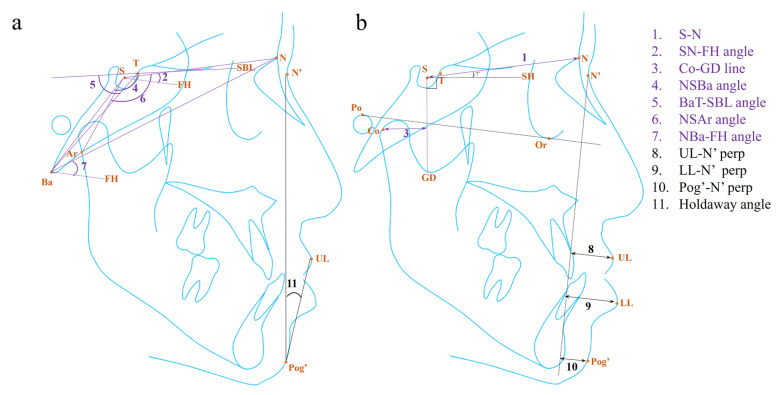
The (**a**) angular, (**b**) linear, and proportional measurements in the cranial base (purple) and soft tissue (black) for diagnosis and treatment of skeletal Class III malocclusion in the existing literature. For the proportional values, only the involved landmark was labeled. The detailed information for the landmark, plane, and measurement is listed in Table S1.

The parameters in different anatomical structures have their own specific patterns. We summarized the parameters for diagnosing and managing skeletal Class III malocclusion in the maxilla, mandible, cranial base, and soft tissue in Table 2 and Table 3 and Figure 2 and Figure 3.

### 3.1. Radiographic Features in the Maxilla

The maxilla is one of the key components of the maxillofacial complex that grows downward and forward, with its growth ending earlier than that of the mandible [19]. In general, maxillary deficiency and proclined upper incisors could be the dentoskeletal characteristics of skeletal Class III malocclusion. The corresponding parameters are related to the skeletal and dental measurements. Notably, although the maxillary measurement has been identified in various domains, it is relatively not a decisive factor and should be considered together with the assessment of other anatomical structures, especially the mandible.

As for the maxillary skeletal parameters, the length and height of the maxilla have been identified as the variables that explained the mild and borderline Class III facial subtypes, which means the maxillary morphology may apparently impact the facial appearance in those Class III cases [12]. In growth prediction, a smaller SNA angle appeared to be a negative predictor for unfavorable growth in children with Class III malocclusion with a threshold value of 79.1 degrees (Figure 2a) [45]. A longer distance between point A and the perpendicular line through point N was identified to represent a tendency for relapse of Class III malocclusion in children receiving early orthopedic treatment, which should be taken into consideration together with a smaller AB-MP angle (Figure 2b) [13,38]. In addition, a longer distance of PH or a more inferior position of PNS was identified as a negative predictor for the growth pattern or the relapse of Class III malocclusion in children and adolescents (Figure 2b) [43,44]. In addition, the PP-SN angle describing the maxillary growth rotation was another predictor of the growth pattern or the relapse of Class III malocclusion in children, where a bigger angle would contribute to a more satisfactory prognosis (Figure 2a) [25,28]. In terms of decision-making, the maxillary dimension was recognized as a predictor for orthognathic surgery need among adult patients with Class III malocclusion based on the ML model, but the model does not provide detailed measurements [34]. Additionally, the Ricketts maxillary depth, which indicates the degree of the maxillary protrusion, was identified as the ML-based predictor for making tooth extraction decisions in patients with different kinds of malocclusions. A smaller angle indicated a greater likelihood of orthodontic extraction need (Figure 2a) [29].

In terms of dental parameters, the maxillary incisor inclination, which was measured by the U1-NA angle, was found to be a predictor of treatment outcomes in Class III children treated with a combination of upper incisor proclination and headgear, where patients with a larger angle might respond poorly to the treatment (Figure 2a) [46]. Besides, the region of maxillary teeth was identified to influence decision-making about surgery using a deep learning model [47]. However, this correlation may not be applicable to all Class III cases, particularly those with mild skeletal discrepancies that do not exhibit compensatory inclination of the upper incisors.

### 3.2. Radiographic Features in the Mandible

The distinct growth of the mandible in skeletal Class III malocclusion has gathered the most interest from researchers and orthodontists for its particularity in the growing pattern and potential. The mandible is displaced downward and forward during growth. Meanwhile, the mandible, along with the maxilla, undergoes complicated rotational growth—internal rotation occurring in the core of the jawbone and external rotation due to bone surface remodeling—which leads to various vertical facial types [58,59]. As early as 1997, Sugawara et al. reviewed longitudinal studies and concluded that the skeletal framework of Class III malocclusion had been established during the pre-pubertal growth period, after which, the increment of annual mandibular growth for Class III patients remained similar to those with a normal face [60]. However, after a decade, another study indicated that the duration of the mandibular growth peak lasted longer in patients with skeletal Class III malocclusion, which can partly account for the larger increment of mandibular growth of Class III patients than those with normal occlusion during growth spurts [61]. The radiographic features in the mandible were summarized, which were related to the mandibular dimension, condyle, chin, mandibular growth rotation, and lower incisors.

The abnormal mandibular dimension is the most direct characteristic in the mandible for Class III malocclusion. The ratios of Ar-Me and Go-Pog to the anterior facial height (AFH) were recognized as the indicators for facial type classification (Figure 2b) [20]. A longer distance of Ar-Gn can help to predict a worse growth pattern in children with skeletal Class III malocclusion (Figure 2b) [43]. A longer distance of Co-Pog was identified as a predictor for the relapse of Class III malocclusion in adolescents after orthopedic therapy (Figure 2b) [44,48]. In addition, a study using ML models recognized the mandibular body dimension as an indicator for decision-making for growth modulation, camouflage orthodontic treatment, or orthognathic surgery [34]. However, this should be combined with other parameters regarding the mandibular growth direction. Recently, a deep learning study has indicated that the mandible, mandibular teeth, and mandibular symphysis were the most influential regions for surgical decision-making, although the study did not provide detailed information on their roles [47]. Apart from parameters or features that directly represent the mandibular dimension, SNB, as an indirect angular parameter, was adopted for facial type classification and predicting the relapse of Class III malocclusion in children undergoing combined orthopedic therapy (Figure 2a) [20,24]. Regarding mandibular ramus height, the distance of Ar-Go was the indicator for facial type classification (Figure 2b) [12,20]. Similarly, the distance of Co-Goi was identified as the predictor for the relapse of Class III malocclusion in the children after orthopedic therapy (Figure 2b) [37,48]. However, whether a longer or shorter distance of Co-Goi can predict the risk of relapse remained controversial, as it should be considered in combination with other mandibular parameters. Another study suggested an increased ratio of Coi-Go to Pogi-Go, with a threshold value of 0.72, was a predictor for the relapse of Class III malocclusion among children after orthodontic treatment (Figure 2b) [23].

The condyle is considered a growth site that largely determines the greatest postnatal growth potential in the mandible [62]. The condylar inclination is critical to the treatment of Class III malocclusion. A larger condylar axis (CondAx)-SBL angle has been associated with the increased stability of treatment outcomes in patients who received removable mandibular retractor therapy (Figure 2a) [36]. It could be explained by a recent study that a larger CondAx-SBL angle may represent an upward-forward inclination of the condyle which is in accordance with the expected change induced by the functional treatment [63]. In addition, the CondAx-MP angle is another predictor for the treatment outcome, where a bigger angle hinted at a larger possibility of relapse in children under rapid maxillary expansion followed by maxillary protraction with a facemask with a cut-off point at 147.8 degrees (Figure 2a) [50]. However, on the contrary, another study found that a smaller CondAx-MP angle may represent poorer treatment stability [5]. The inconsistency between the two studies might be related to the variations in the facial subtypes included. 

In terms of the chin, the FP-SN angle and FP-FH angle were identified as the variables for the severe mandibular prognathic facial subtype (Figure 2a) [12]. The chin morphology measured by the MP-PogId angle was recognized for facial type classification, and a smaller GnGoi-BPog angle was a negative predictor for the relapse among adolescents receiving orthopedic treatment (Figure 2a) [11,20].

As one of the mechanisms of mandibular growth patterns mentioned above, internal rotation is one type of mandibular growth rotation. The internal rotation is composed of matrix rotation occurring around the center of the condyle, as well as intra-matrix rotation around the center inside the mandible [59]. As for the matrix rotation-related parameters, the distances of S-Ar, B-N, and Pog-N have been identified as the indicators for the classification of severe and mild Class III facial subtypes (Figure 2b) [12]. A larger distance of ArH was a negative predictor for the deterioration of Class III malocclusion during growth (Figure 2b) [43]. Additionally, a smaller GZN angle, defined by the angle between the ramus plane and the SN plane, together with a superiorly positioned Go, can help to predict a larger possibility of relapse of Class III malocclusion in children after early orthopedic treatment (Figure 2a) [24,44,51]. In the measurement for the intra-matrix rotation, a larger gonial angle was a significant parameter for a greater likelihood of relapse in children receiving combined orthopedic therapy (Figure 2a) [11,22,23,39,48,51]. A larger lower gonial angle which is divided by the N-Go line, was identified as an indicator of surgical need in adult patients [30]. Notably, the articular angle, the SArGo angle, was the parameter that described both the matrix and intra-matrix rotation (Figure 2a). It was identified as the variable explaining the treatment outcome of Class III children under the facemask treatment, where children with a smaller angle tended to experience Class III malocclusion relapse [27]. Considering mandibular rotation, Class III patients with prognathic mandibles and long faces are more likely to be treated with orthognathic surgery according to the ML model [53].

In the measurement of lower incisors, the distances of L1-FH, L1-NB, and L1-GoGn were the variables that explained the maxillary deficiency and high-angle subtype where there was apparently a compensatory inclination of the lower incisors (Figure 2b) [12]. A longer distance of L1H could predict the growth worsening in children with Class III malocclusion (Figure 2b) [43]. For the treatment, a smaller L1-MP angle and a larger L1-FH angle and L1-OP angle could assist in predicting Class III malocclusion relapse in children after various orthopedic therapies (Figure 2a) [25,49,51]. In addition, the distance of L1-NB was found to account for decision-making about tooth extraction according to the ML model. However, the study did not provide detailed information about how the distance predicted the orthodontic extraction mode due to the limitation of the ML model (Figure 2b) [52]. The lower incisor inclination also played a role in predicting the need for orthognathic surgery. A smaller L1-MP angle was the indicator for orthognathic surgical need in adult patients (Figure 2a) [32,35]. As for the treatment plan for the surgery, Chen et al. found that orthognathic surgery patients with a smaller L1-MP angle had a higher tendency to undergo the surgery-first approach with a threshold value of 76.1 degrees (Figure 2a) [54]. The authors supposed that the characteristic appeared more in Class III patients with a high angle, who generally had less dental crowding and compensation. Recently, Zhang et al. found that the angle between the axis of the mandibular symphysis and L1 should be taken into consideration during tooth decompensation treatment before orthognathic surgery, in case the tooth root came out of the alveolar bone [55].

### 3.3. Radiographic Features in the Cranial Base

As the “engine” of maxillary growth, the cranial base matures quite earlier compared with other maxillofacial bones, indirectly determining the shape and position of the maxillomandibular complex [64]. The parameters identified in this region mainly consist of the cranial base length and deflection.

The anterior cranial base length has been found to decrease in patients with Class III malocclusion, especially in Japanese and Chinese patients compared with British Caucasian patients due to their own genetic characteristics [65,66]. Then, the reduction of the anterior cranial base length may impact maxillary position, leading to maxillary deficiency [67]. In the diagnostic application, the anterior cranial base, i.e., the distance of S-N, was one of the variables for the mildly mandibular prognathic subtype in Class III patients (Figure 3b) [12]. In addition, it was also identified in relation to surgical decision-making, where the decreased length represented a greater demand for orthognathic surgery among Class III adult patients [30].

The cranial base deflection can be measured by the angles formed by the anterior or posterior cranial base and other reference planes. The SN-FH angle was identified as an indicator for the borderline Class III malocclusion subtype (Figure 3a) [12]. However, as nasion is not part of the cranial base, Seetala et al. proposed that the point of sphenoidale (Se), the intersection point between the sphenoid and ethmoid bones in the lateral cephalograms, played a role in measuring the anterior cranial base inclination [67]. Meanwhile, they observed a smaller cranial base angle (NSBa angle and SeSBa angle) and posterior cranial–base inclination (FH-SBa angle) in patients with Class III malocclusion, which means that the point of Ba was located more anteriorly in Class III patients [67]. It will then give rise to a more anterior position of the points of Co, Ar and even the whole mandible, leading to the deterioration of the discrepancy in the jaw relationship [31,48]. Similarly, studies identified a shorter distance between Co and a vertical line through S (GD line), a smaller NSBa angle, and a larger BaT-SBL angle as the predictors for the relapse of Class III malocclusion in children under orthopedic treatment (Figure 3a,b) [11,37,48]. Additionally, a smaller NSAr angle and a greater NBa-FH angle were found to be associated with a higher need for orthognathic surgery in Class III adult patients through discriminant analysis and the ML model (Figure 3a) [8,31].

### 3.4. Radiographic Features in the Soft Tissue

The soft tissues, such as the tongue and masticatory muscles, are closely related to the maxillofacial skeleton [68]. Although the radiographic features of bony tissues are significant for the diagnosis and treatment of malocclusion, the attention of orthodontists should not be completely shifted from the facial soft tissue to the skeletal structure, as facial soft tissue harmony is one of the goals of orthodontic treatment [69]. The parameters identified in this domain are mainly related to the position of the lips and the Holdaway angle.

The identified parameters describing the lip position mainly involved the sagittal position of the upper and lower lips. The distance between the point of UL and the perpendicular line through the point of N’ (N’ perp) was identified as the parameter explaining the borderline Class III malocclusion subtype, probably due to its availability for the detection of a mildly concave mid-face (Figure 3b) [12]. Besides, the distances of N’ perp-LL and N’ perp-Pog’ were the indicators explaining the variance of the subtypes of mandibular prognathism and a long face (Figure 3b) [12]. 

The Holdaway angle is a significant parameter, especially for decision-making regarding orthognathic surgery. Proposed by Holdaway in 1983, it refers to the angle formed by the harmony line (H line) and N’-Pog’ in order to measure the prominence of the upper lip and chin (Figure 3a) [70]. Some researchers believed that this soft tissue parameter could be more helpful in assisting treatment planning than the bony ones, especially in borderline Class III malocclusion cases where the facial aesthetics might be more critical than the skeletal discrepancy [33]. A smaller Holdaway angle was identified as an indicator for the need for orthognathic surgery in adult patients with Class III malocclusion, with the cut-off value ranging from 7.2 to 12 degrees in different studies [33,56,57]. The variation could be due to diverse races and inclusion criteria for Class III malocclusion. Additionally, it was also verified by the ML model as an indicator for surgical decision-making [35].

## 4. Radiographic Features in 3D Images of Skeletal Class III Malocclusion

Given the irregular shape and the intricate anatomical structure of the maxillofacial skeleton, three-dimensional (3D) imaging tends to be a more satisfactory approach for detecting oral and maxillofacial diseases, as well as the discrepancies in dental alignment and/or the jaws. However, with the relatively late emergence of 3D imaging technologies, only a few radiological features have been observed in Class III malocclusion cases based on CBCT and MRI until now, in contrast to numerous parameters identified in traditional 2D images. As 3D imaging technologies continue to advance, it is expected that more comprehensive and accurate parameters will be discovered, leading to the improved diagnosis and treatment of Class III malocclusion. We have summarized the representative features for Class III malocclusion in 3D images to provide a reference for future research (Table 4).

### 4.1. Radiographic Features in CBCT

The different locations of the mandibular canal (MC) in relation to the mandible in patients with Class III malocclusion have been preliminary studied. Huang et al. measured the position of MC in the inner and mid-posterior dimensions and did not observe significant differences in patients with various kinds of dentofacial relationships [71]. Nevertheless, F. Kalabalik later measured the 3D location of MC and found a more buccal, superior and forward position of MC in patients with Class III malocclusion compared with those with Class Ⅰ malocclusion. This may serve as a reference to reduce the risk of neurosensory disturbance during the orthognathic surgery [73].

Research has also focused on the specific features of condyles. Petra et al. demonstrated that Class III subjects had longer and larger condyles with higher antero–posterior and medio–lateral inclination angles based on CBCT [63]. The hyperdivergent subjects had smaller condyles with higher antero–posterior inclination angles, which further illustrates a strong association between the shape and the growth direction of condyles and the whole mandible [63]. Additionally, Kim et al. measured condylar bone densities in adolescents with different types of malocclusions and observed that the cortical, cancellous, and total bone densities increased as the ANB angle increased and the postero–anterior facial height ratio decreased [72]. It hinted that condylar bone densities could be candidate predictors for growth patterns in patients with different kinds of malocclusion.

Although CBCT is expected to aid in the diagnosis and treatment of malocclusion, the radiation protection of CBCT should be seriously taken into consideration in clinical practice. As described in the previous study, the radiation dose of a CBCT is about 3–6 times a panoramic radiograph (OPG), 8–14 times a postero–anterior cephalogram (PA), and 15–26 times a lateral cephalogram [78]. Hence, the ALARA (As Low As Reasonably Achievable) principle should always be followed by orthodontists and radiologists [79].

### 4.2. Radiographic Features in MRI

Studies have suggested that the temporomandibular joint (TMJ) disk and tongue may show some adaptive or even pathological changes in relation to the Class III malocclusion, which could be detected using MRI. It was reported that the displacement and abnormal shape of the TMJ disk were more common in patients with Class III malocclusion compared with those with Class Ⅰ malocclusion, while whether the vertical facial type was related to TMJ disk displacement remained controversial [75,76,77]. Additionally, in accordance with the growth pattern of the maxillofacial skeleton in Class III malocclusion cases, the posterior portion of the dorsal tongue and the tip of the tongue were reported to be positioned more inferiorly and anteriorly, respectively [74]. The tongue movement during the deglutition stage was also found to be different in patients with skeletal Class III malocclusion [74]. Future research should take advantage of MRI’s ability to detect dynamic changes in soft tissue to better elucidate their relationship with the maxillofacial skeleton and soft tissue in the context of skeletal Class III malocclusion.

## 5. Current States and Future Prospects

The development of radiology has greatly contributed to the diagnosis and treatment of skeletal Class III malocclusion. In this review, we provided a comprehensive summary of the significant parameters on 2D cephalograms for inter-maxillary relationship and various anatomical structures. Additionally, we synthesize the characteristics related to skeletal Class III malocclusion in 3D images based on the limited number of studies available. These radiographic features have the potential to assist in classifying facial type, predicting growth, making decisions regarding tooth extraction or orthognathic surgery, and guiding clinical practices. However, several limitations in current research still exist that may hinder the practical application of these features in diagnosing or treating Class III malocclusion.

In addition to the lateral cephaolograms, CBCT and MRI images, the OPG, and PA are commonly used in orthodontic clinical practice. Nevertheless, since the OPG provides a panoramic image of the teeth, mandible, and maxilla, and the PA is the cephalogram taken from the frontal view, both of them are not sensitive to detect the Class III malocclusion subtype which is characterized by the sagittal discrepancy. Up to now, no predictors for the diagnosis and treatment of skeletal Class III malocclusion have been identified in the OPG and PA.

The significant parameters identified in different studies often share little similarity, which may undermine their value in clinical application and generalization. This discrepancy might be associated with the following aspects. First, many studies had an insufficient sample size, especially longitudinal studies for growth prediction. Second, the standards for evaluating growth patterns or treatment outcomes differed among studies. In the cases of decision-making for tooth extraction or orthognathic surgery, it was the experts’ opinions that served as the grouping standards, potentially introducing bias. Third, variations in the quantity and the types of input values across studies, together with potential examiner measurement errors, could affect the power of identifying the significant parameters. Finally, the conventional statistical models or the ML algorithms performed in each study were not always consistent. These reasons make it difficult to objectively compare and apply those significant variables from various studies.

Despite plenty of identified parameters for the diagnosis and treatment of Class III malocclusion, the weight or priority of each variable in different structures remains unclear. Each parameter has its own advantage and disadvantage, making it difficult to select valuable indicators or a complete variable set from the candidate parameters to assist in the precise diagnosis or the treatment outcome prediction. In addition, the performance of the models in the diagnosis or treatment of Class III malocclusion was not always satisfactory, especially in borderline Class III cases [52]. Thus, the constructed models may not completely assist less-experienced orthodontists in practice.

Several ML models have been developed in orthodontics to identify relevant variables with greater accuracy compared to conventional statistical models [17,80,81,82]. Recently, two studies have developed deep learning models to predict the need for orthognathic surgery using lateral cephalograms [47,83]. Although these deep learning models obtained favorable prediction accuracy, interpreting their prediction outcomes is challenging due to their “black box” nature, making the decision-making process difficult to explain [17]. Most recently, it has been raised that quantitative textural imaging analysis could be utilized to predict the growth of specific organs or the progression of lesions [84,85]. The integration of ML and quantitative imaging feature analysis (i.e., radiomics) may potentially advance the personalized diagnosis and treatment of Class III malocclusion, enhancing the understanding of the underlying mechanisms of maxillofacial growth.

## 6. Conclusions

Radiographic features in 2D and 3D radiographic images provide valuable insight for the diagnosis and treatment of skeletal Class III malocclusion. Various parameters related to the inter-maxillary relationship and different anatomical structures contribute to the accurate diagnosis and prediction of facial growth or treatment outcomes, decision-making for tooth extraction and surgery, and guiding clinical practices. However, there are still some challenges to adopting these parameters into clinical practices due to their high diversity. With the improvement in generalizability and analytic approaches, including ML and radiomics, the significant radiographic features may become more informative for orthodontists to provide precise and personalized dental services.

## Figures and Tables

**Table 1:** Studies evaluating the association of inter-maxillary relationship with radiographic features on cephalograms using conventional statistical methods or machine learning approaches.

Author (Year)	Application	Sample Size	Inclusion	Treatment	Judgment Criteria	Type of Outcome	Period	Architecture	Input	Significant Output	Trend for Poorer Result/Extraction/Surgery Need (Cut-Off Point)
Sagittal dimension											
Kim C (1995) [20]	Facial type	46	Around 8-year-old children with skeletal Class Ⅲ malocclusion	CC	Cluster analysis	5 groups with different effect of chin-cup therapy	Around 6 years	Discriminant analysis	17 cephalometric variables	ANB angle	None
Chi Bui (2006) [12]	Facial type	309	Patients with skeletal Class Ⅲ malocclusion	None	Cluster analysis	5 clusters representing distinct subphenotypes	Not mentioned	Cluster and principal component analyses	67 cephalometric variables	ANB angle, unit length difference, interincisal angle, Wits appraisal	None
Akane Ueda (2023) [21]	Facial type	220	Adults with skeletal Class Ⅰ or Ⅱ or Ⅲ malocclusion	Orthodontic treatment	Dentist	9 maxillofacial morphology classifications	Not mentioned	Machine learning (random forest) (top 3 features with the highest importance)	9 cephalometric variables and nonradiographic variables	ANB angle	None
A. Stensland (1988) [11]	Growth prediction	91	4 to 9-year-old children with normal jaw relationship or skeletal Class Ⅲ malocclusion	Retractor + CC	Positive overjet	Success, relapse	5 to 18 months	Discriminant analysis	35 cephalometric variables	U1-L1 angle	Larger
Khatoon Tahmina (2000) [22]	Growth prediction	56	Children with skeletal Class Ⅲ malocclusion	CC + FIX	Treatment outcome or the occlusal status at the end of treatment after pubertal growth	Success, relapse	9 years on average	Discriminant analysis	20 cephalometric variables	NAPog angle	Larger
Andrej Zentner (2001) [23]	Growth prediction	80	Children with Class Ⅲ base relationship	FUN + FIX	Change of the peer assessment rating index	Greatly improved, improved, worse/no difference	5 years on average	Regression analysis	23 cephalometric variables	Co-A/Co-Gn, net sum of maxillary difference and mandibular difference	Smaller (0.74), None
Adolfo Ferro (2003) [24]	Growth prediction	52	Children with skeletal Class Ⅲ malocclusion	Splints + Class III elastics + CC	Positive overjet and overbite	Success, relapse	9 years on average	*T*-test	20 cephalometric variables	Wits appraisal, ANB angle	Smaller, smaller
Gabriele Schuster (2003) [25]	Growth prediction	88	Children with skeletal Class Ⅲ malocclusion	CC + HG + FIX	A surgery need based on 3 experienced orthodontists	Success, relapse	At least 4 years	Discriminant analysis and regression analysis	20 cephalometric variables	Wits appraisal	Smaller
Peter Ngan (2004) [26]	Growth prediction	40	Children with skeletal Class Ⅲ malocclusion	RME + HG	A positive overjet of greater than 1 mm at the follow-up visit	Success, relapse	A minimum of 3 years	*T*-test	None	Growth Treatment Response Vector (GTRV)	Smaller (0.38)
Yoon Jeong Choi (2017) [27]	Growth prediction	59	Around 9-year-old children with skeletal Class Ⅲ malocclusion	FM	Overjet, overbite, and the acceptable facial profile agreed by three orthodontists	Success, relapse	Until the growth completion	Logistic regression analysis	34 cephalometric variables	Wits appraisal	Smaller
Pietro Auconi (2021) [28]	Growth prediction	104	Children with skeletal Class Ⅲ malocclusion	None	The ANB angle, the Wits appraisal, and the ratio of Co-Gn/Co-A	Very serious growing subjects, mild subjects	At least one year and 6 months. About 3 years on average	Logistic regression analysis on case-based reasoning	15 cephalometric variables	Wits appraisal	Smaller
Alberto Del Real (2022) [29]	Extraction-decision	214	Patients with skeletal Class Ⅰ or Ⅱ or Ⅲ malocclusion	Comprehensive orthodontic treatment in permanent dentition	Dentist	With or without orthodontic extraction	Not mentioned	Machine learning (sequential minimal optimization algorithm)	42 cephalometric variables and nonradiographic inputs	Wits appraisal	Larger
Angelika Stellzig-Eisenhauer (2002) [30]	Surgery-decision	175	Adults with skeletal Class Ⅲ malocclusion	Surgery or none-surgery treatment	Dentist	Nonsurgery group and surgery group	Not mentioned	The discriminant function model	20 cephalometric variables	Wits appraisal, ratio of anteroposterior length of maxilla to anteroposterior length of mandible	Smaller, smaller
Janka Kochel (2011) [31]	Surgery-decision	69	Adults with skeletal Class Ⅲ malocclusion	Surgery or nonsurgery treatment	Dentist	Nonsurgery group and surgery group	Not mentioned	A discriminant analysis	19 cephalometric variables	Wits appraisal, ratio of anteroposterior length of maxilla to anteroposterior length of mandible	Smaller, smaller
P Martinez (2017) [32]	Surgery-decision	156	Adults with skeletal Class Ⅲ malocclusion	Surgery or nonsurgery treatment	Dentist	Nonsurgery group and surgery group	Not mentioned	The Student *t*-testand ANOVA	9 cephalometric variables	Wits appraisal, U1-L1 angle	Smaller, larger
Sara Eslami (2018) [33]	Surgery-decision	65	Adults with moderate skeletal Class Ⅲ malocclusion	Surgery or nonsurgery treatment	Dentist	Orthodontic treatment with or without orthognathic surgery	Not mentioned	Stepwise discriminant analysis	24 cephalometric variables	Wits appraisal	Smaller (−5.8 mm)
Jahnavi Prasad (2022) [34]	Surgery-decision	700	10 to 30-year-old patients with skeletal Class Ⅰ or Ⅱ or Ⅲ malocclusion	Growth modulation, camouflage, or jaw surgery	Dentist	Extractions options in Class Ⅰ malocclusion; Growth modulation, camouflage and jaw surgery in Class Ⅱ and Ⅲ malocclusion	Not mentioned	Machine learning (7 kinds of algorithm) (top 10 parameters with the highest contribution)	33 cephalometric variables and nonradiographic inputs	Wits appraisal, beta angle	None
Hunter Lee (2022) [35]	Surgery-decision	196	Skeletal Class III patients	Surgery or nonsurgery treatment	Dentist	Nonsurgery group and surgery group	Not mentioned	Machine learning (random forest and logistic regression) (top 3 features with the highest importance scores in the specific algorithm)	60 cephalometric variables and nonradiographic inputs	Wits appraisal	Smaller
Samim Taraji (2023) [8]	Surgery-decision	182	Adults with skeletal Class Ⅲ malocclusion	Surgery or nonsurgery treatment	Dentist	Nonsurgery group and surgery group	11 to 70 months	Machine learning (XG boost analysis) (with top 3 weights in XGBoost analysis)	40 cephalographic variables and nonradiographic inputs	Wits appraisal, A-Ar/Gn-Ar	Smaller, smaller
Vertical dimension											
Chi Bui (2006) [12]	Facial type	309	Patients with skeletal Class Ⅲ malocclusion	None	Cluster analysis	5 clusters representing distinct subphenotypes	Not mentioned	Cluster and principal component analyses	67 cephalometric variables	SN-GoGn, total facial height, LFH, upper facial height, posterior facial height, upper first molar-mandibular plane height	None
Akane Ueda (2023) [21]	Facial type	220	Adults with skeletal Class Ⅰ or Ⅱ or Ⅲ malocclusion	Orthodontic treatment	Dentist	9 maxillofacial morphology classifications	Not mentioned	Machine learning (random forest) (top 3 features with the highest importance)	9 cephalometric variables and nonradiographic inputs	MP-FH angle, MP-SN angle	None
Lorenzo Franchi (1997) [36]	Growth prediction	45	Around 5-year-old children with skeletal Class Ⅲ malocclusion due to mandibular protrusion	Removable mandibular retractor treatment	The concomitant presence of Class III permanent molar relationship, Class Ill permanent canine relationship and anterior crossbite of at least one incisor was defined as failure of treatment.	Success, relapse	9 years on average	Discriminant analysis	20 cephalographic variables, and nonradiographic inputs	PP-MP angle	Larger
Tiziano Baccetti (2004) [37]	Growth prediction	42	Children with skeletal Class Ⅲ malocclusion	RME + FM	The presence of Class III permanent molar relationship and negative overjet were defined as unsuccessful.	Success, relapse	6.5 years on average	Discriminant analysis	19 cephalometric variables and nonradiographic inputs	MP-SBL angle	Larger
Young-Min Moon (2005) [38]	Growth prediction	45	Children with Class Ⅲ malocclusion	CC + FIX	Overjet, overbite, and the orthognathic surgery need	Success, uncertain, relapse	At least 2 years after the end of treatment	Discriminant analysis	20 cephalometric variables	AB-MP angle	Smaller
Ikue Yoshida (2006) [39]	Growth prediction	32	Children with skeletal Class Ⅲ malocclusion	FM + CC + FIX	Status of the anterior bite and molar and canine relationships	Success, relapse	About 7 years on average	Discriminant analysis and regression analysis	20 cephalometric variables	ANS-Me	Larger
Bo-Mi Kim (2009) [13]	Growth prediction	38	Children with skeletal Class Ⅲ malocclusion	CC/FM + FIX	The favorable occlusal status with a normal overbite and overjet	Success, relapse	9 years on average	Feature wrapping method and discriminant analysis	46 cephalometric variables	AB-MP angle	Smaller
Yoon Jeong Choi (2017) [27]	Growth prediction	59	9-year-old children with skeletal Class Ⅲ malocclusion	FM	Overjet, overbite and the acceptable facial profile agreed by three orthodontists	Success, relapse	Until the growth completion	Logistic regression analysis	34 cephalometric variables	AB-MP angle	Smaller
Alberto Del Real (2022) [29]	Extraction-decision	214	Patients with skeletal Class Ⅰ or Ⅱ or Ⅲ malocclusion	Comprehensive orthodontic treatment in permanent dentition	Dentist	With or without orthodontic extraction	Not mentioned	Machine learning (a multilayer perceptron algorithm and sequential minimal optimization algorithm)	42 cephalometric variables and nonradiographic inputs	Ricketts facial axis	Larger
Samim Taraji (2023) [8]	Surgery-decision	182	Adults with skeletal Class Ⅲ malocclusion	Surgery or nonsurgery treatment	Dentist	Nonsurgery group and surgery group	11 to 70 months	Machine learning (XG boost analysis) (with top 3 weights in XGBoost analysis)	40 cephalographic variables and nonradiographic inputs	PP-MP angle and MP angle	Larger, larger

RME, rapid maxillary expansion; FM, facemask; HG, headgear; CC, chin-cup; FUN, functional appliance; FIX, fixed appliance.

**Table 2:** Studies evaluating the association of maxilla and mandible with radiographic features on cephalograms using conventional statistical methods or machine learning approaches.

Author (Year)	Application	Sample Size	Inclusion	Treatment	Judgment Criteria	Type of Outcome	Period	Architecture	Input	Significant Output	Trend for Poorer result/Extraction/Surgery Need (Cut-Off Point)
Maxilla dimension											
Chi Bui (2006) [12]	Facial type	309	Patients with skeletal Class Ⅲ malocclusion	None	Cluster analysis	5 clusters representing distinct subphenotypes	Not mentioned	Cluster and principal component analyses	67 cephalometric variables	Maxillary unit length, A-N perp	None
Elham S. J. Abu Alhaija (2003) [43]	Growth prediction	115	Adolescents with skeletal Class Ⅲ malocclusion	None	Patients whose changes in Wits measurements were over 2.5 mm are defined poor growers.	Good and bad growers	3.7 years on average. At least one year.	Hierarchical cluster analysis and discriminant function analysis (top 5 highest discriminant function coefficients)	60 cephalometric variables	PH	Larger
Gabriele Schuster (2003) [25]	Growth prediction	88	Children with skeletal Class Ⅲ malocclusion	CC + HG + FIX	A surgery need based on 3 experienced orthodontists	Success, relapse	At least 4 years	Discriminant analysis and regression analysis	20 cephalometric variables	PP-SN angle	Smaller
Young-Min Moon (2005) [38]	Growth prediction	45	Children with Class Ⅲ malocclusion	CC + FIX	Overjet, overbite, and the orthognathic surgery need	Success, uncertain, relapse	At least 2 years after the end of treatment	Discriminant analysis	20 cephalometric variables	A-N perp	Larger
Andrew P. Wells (2006) [44]	Growth prediction	41	Children with skeletal Class Ⅲ malocclusion	RME + FM	The negative overjet was defined as failure	Success, relapse	At least 5 years after treatment	Discriminant analysis	24 cephalometric variables	Vertical coordinate of PNS	Smaller
Bo-Mi Kim (2009) [13]	Growth prediction	38	Children with skeletal Class Ⅲ malocclusion	CC/FM + FIX	The favorableocclusal status with a normal overbite and overjet	Success, relapse	9 years on average	Feature wrapping method and discriminant analysis	46 cephalometric variables	A-N perp	Larger
Pietro Auconi (2017) [45]	Growth prediction	91	Untreated Class III children	None	Based on the difference betweenCo–Gn and Co–A	Unfavorable growers and favorable growers	5 years on average	Classification trees	11 cephalometric variables	SNA angle	Smaller (79.1 degrees)
Marco Nassar Blagitz (2020) [46]	Growth prediction	36	Patients with unilateral or bilateral canine Class III malocclusion or with skeletal deformities	FIX	Patients with relapse were defined with edge-to-edge or incisor crossbite and/or Class III canine relationship after treatment	Success, relapse	At least 3 years after treatment	Multivariate Poisson regression analysis	7 cephalometric variables and other nonradiographic inputs	U1-NA angle	Larger
Pietro Auconi (2021) [28]	Growth prediction	104	Children with skeletal Class Ⅲ malocclusion	None	The worsening of ANB angle and the Wits appraisal, as well as the ratio of Co-Gn/Co-A	Very serious growing subjects, mild subjects	At least one year and 6 months. About 3 years on average	Logistic regression analysis on case-based reasoning	15 cephalometric variables	PP-SN angle	Smaller
Alberto Del Real (2022) [29]	Extraction-decision	214	Patients with skeletal Class Ⅰ or Ⅱ or Ⅲ malocclusion	Comprehensive orthodontic treatment in permanent dentition	Dentist	With or without orthodontic extraction	Not mentioned	Machine learning (a multilayer perceptron algorithm and sequential minimal optimization algorithm)	42 cephalometric variables and nonradiographic inputs	Ricketts maxillary depth	Smaller
Ki-Sun Lee (2020) [47]	Surgery-decision	333	Patients with Class Ⅰ or Ⅱ or Ⅲ malocclusion with or without skeletal discrepancies	Surgery or nonsurgery treatment	Dentist	Nonsurgery group and surgery group	Not mentioned	Machine learning (Modified-Alexnet, MobileNet and Resnet50)	50 cephalometric variables	Maxillary teeth	None
Jahnavi Prasad (2022) [34]	Surgery-decision	700	10 to 30-year-old patients with skeletal Class Ⅰ or Ⅱ or Ⅲ malocclusion	Growth modulation, camouflage, or jaw surgery	Dentist	Extractions options in Class Ⅰ malocclusion; Growth modulation, camouflage and jaw surgery in Class Ⅱ and Ⅲ malocclusion	Not mentioned	Machine learning (7 kinds of algorithm) (top 10 parameters with the highest contribution)	33 cephalometric variables and nonradiographic inputs	Maxillary dimension	None
Mandibular dimension											
Kim C (1995) [20]	Facial type	46	Around 8-year-old children with skeletal Class Ⅲ malocclusion	CC	Cluster analysis	5 groups with different effect of chin-cup therapy	Around 6 years	Discriminant analysis	17 cephalometric variables	SNB angle, SNP angle, MP-PogId angle, Ar-Me/AFH, Go-Pog/AFH, Ar-Go, GZN angle	None
Chi Bui (2006) [12]	Facial type	309	Patients with skeletal Class Ⅲ malocclusion	None	Cluster analysis	5 clusters representing distinct subphenotypes	Not mentioned	Cluster and principal component analyses	67 cephalometric variables	S-Ar, FP-SN angle, FP-FH angle, B-N, Pog-N, L1-NB, L1 protrusion, L1-GoGn, L1-FH, mandibular unit length, ramus height	None
A. Stensland (1988) [11]	Growth prediction	91	4 to 9-year-old children with normal jaw relationship or skeletal Class Ⅲ malocclusion	Retractor + CC	Positive overjet	Success, relapse	5 to 18 months	Discriminant analysis	35 cephalometric variables	Pronounced mandibular prognathism, gonial angle, BPog-GnGoi angle	More apparent, larger, smaller
Lorenzo Franchi (1997) [36]	Growth prediction	45	Around 5-year-old children with skeletal Class Ⅲ malocclusion due to mandibular protrusion	Removable mandibular retractor treatment	The concomitant presence of Class III permanent molar relationship, Class Ill permanent canine relationship and anterior crossbite of at least one incisor was defined as failure of treatment.	Success, relapse	9 years on average	Discriminant analysis	20 cephalographic variables, and nonradiographic inputs	CondAx-SBL angle	Smaller
Khatoon Tahmina (2000) [22]	Growth prediction	56	Children with skeletal Class Ⅲ malocclusion	CC + FIX	Treatment outcome or the occlusal status at the end of treatment after pubertal growth	Success, relapse	9 years on average	Discriminant analysis	20 cephalometric variables	Gonial angle, ramus plane-SN plane angle	Larger, smaller
Andrej Zentner (2001) [23]	Growth prediction	80	Children with Class Ⅲ base relationship	FUN + FIX	Change of the peer assessment rating index	Greatly improved, improved, worse/no difference	5 years on average	Regression analysis	23 cephalometric variables	Go-Coi/Go-Pogi, gonial angle	Larger (0.72), larger
Elham S. J. Abu Alhaija (2003) [43]	Growth prediction	115	Adolescents with skeletal Class Ⅲ malocclusion	None	Patients whose changes in Wits measurements were over 2.5 mm are defined poor growers.	Good and bad growers	3.7 years on average At least one year	Hierarchical cluster analysis and discriminant function analysis (top 5 highest discriminant function coefficients)	60 cephalometric variables	Ar-Gn, ArH, ArP (projected Ar on SH) –GnP (projected Gn on SH), LiH	Larger, larger; larger, larger
Gabriele Schuster (2003) [25]	Growth prediction	88	Children with skeletal Class Ⅲ malocclusion	CC + HG + FIX	A surgery need based on 3 experienced orthodontists	Success, relapse	At least 4 years	Discriminant analysis and regression analysis	20 cephalometric variables	L1-MP angle	Smaller
Adolfo Ferro (2003) [24]	Growth prediction	52	Children with skeletal Class Ⅲ malocclusion	Splints + Class III elastics + CC	Positive overjet and overbite	Success, relapse	9 years on average	*T*-test	20 cephalometric variables	SNB angle	Larger
Tiziano Baccetti (2004) [37]	Growth prediction	42	Children with skeletal Class Ⅲ malocclusion	RME + FM	The presence of Class III permanent molar relationship and negative overjet were defined as unsuccessful.	Success, relapse	6.5 years on average.	Discriminant analysis	19 cephalometric variables and nonradiographic inputs	Co–Goi	Larger
Matthew A. Ghiz (2005) [48]	Growth prediction	64	Children with skeletal Class Ⅲ malocclusion	RME + FM	A positive overjet and a Class Ⅰ molar relationship	Success, relapse	At least 3 years after treatment	Regression analysis	18 cephalometric variables	Co–Goi, Co–Pog, gonial angle	Smaller, larger, larger
Young-Il Ko (2004) [49]	Growth prediction	40	Children with skeletal Class III malocclusion solely due to mandibular overgrowth	CC + FIX	A good facial profile, positive overbite and overjet, and Class I canine and molar occlusal relationship without severe facial and dental asymmetry were the criteria for good retention.	Success, relapse	9 years on average.	*T*-test (the most significant features (*p* < 0.001) scores in the specific algorithm)	55 cephalometric variables	L1-OP angle	Larger
Andrew P. Wells (2006) [44]	Growth prediction	41	Children with skeletal Class Ⅲ malocclusion	RME + FM	The negative overjet was defined as failure	Success, relapse	At least 5 years after treatment	Discriminant analysis	24 cephalometric variables	Vertical position of Go, mandibular unit length	Smaller, larger
Ikue Yoshida (2006) [39]	Growth prediction	32	Children with skeletal Class Ⅲ malocclusion	FM + CC + FIX	Status of the anterior bite and molar and canine relationships	Success, relapse	About 7 years on average	Discriminant analysis and regression analysis	20 cephalometric variables	Gonial angle	Larger
Daniele Nóbrega Nardoni (2015) [5]	Growth prediction	26	Children who had maxillary deficiency and/or mandibular prognathism with Class I or Class III malocclusion in mixed dentition	RME + FM	Subjective facial analysis by the evaluators and the self-perception from patients	Success, relapse	6 years and 10 months on average	Discriminant analysis	18 cephalometric variables	LAFH combined with the CondAx-MP angle	Larger, smaller
Yoon Jeong Choi (2017) [27]	Growth prediction	59	9-year-old children with skeletal Class Ⅲ malocclusion	FM	Overjet, overbite, and the acceptable facial profile agreed by three orthodontists	Success, relapse	Until the growth completion	Logistic regression analysis	34 cephalometric variables	SArGo angle	Smaller
Bernardo Quiroga Souki (2020) [50]	Growth prediction	101	7 to 9-year-old children with skeletal Class Ⅲ malocclusion	RME + FM	The combination of occlusion and lateral cephalograms	Success, relapse	At least 5 years	Bivariate logistic regression analysis	24 cephalometric variables and nonradiographic inputs	CondAx-MP angle	Larger (147.8 degrees)
Yasuko Inoue (2021) [51]	Growth prediction	75	Children with skeletal Class Ⅲ malocclusion	RME + FM	Positive overjet	Success, relapse	About 6 years on average	Logistic regression analysis	13 cephalometric variables and nonradiographic inputs	SN-ramus plane angle, gonial angle, FH-L1 angle	Smaller, larger, larger
Lily Etemad (2021) [52]	Extraction-decision	838	Patients with Class Ⅰ or Ⅱ or Ⅲ malocclusion	FIX	Dentist	With or without orthodontic extraction	Not mentioned	Machine learning (random forest)	22 cephalometric parameters and nonradiographic inputs	L1-NB	None
Angelika Stellzig-Eisenhauer(2002) [30]	Surgery-decision	175	Adults with skeletal Class Ⅲ malocclusion	Surgery or nonsurgery treatment	Dentist	Nonsurgery group and surgery group	Not mentioned	The discriminant function model	20 cephalometric variables	Lower gonial angle	Larger
P Martinez (2017) [32]	Surgery-decision	156	Adults with skeletal Class Ⅲ malocclusion	Surgery or nonsurgery treatment	Dentist	Nonsurgery group and surgery group	Not mentioned	The Student *t*-testand ANOVA	9 cephalometric variables	L1-MP angle	Smaller
Ki-Sun Lee (2020) [47]	Surgery-decision	333	Patients with Class Ⅰ or Ⅱ or Ⅲ malocclusion with or without skeletal discrepancies	Surgery or nonsurgery treatment	Dentist	Nonsurgery group and surgery group	Not mentioned	Machine learning (Modified-Alexnet, MobileNet and Resnet50)	50 cephalometric variables	Mandibular teeth, mandibular symphysis and mandible	None
Pegah Khosravi-Kamrani (2022) [53]	Surgery-decision	148	7 to 25 –year-old patients with skeletal Class Ⅲ malocclusion	Surgery or nonsurgery treatment	Straight profile, overjet, overbite, absence of anterior or posterior crossbite	Success, relapse	Not mentioned	Machine learning analysis	67 cephalometric variables	Patients with mandibular prognathic and long face experienced higher likelihood of treatment failure.	None
Jahnavi Prasad (2022) [34]	Surgery-decision	700	10 to 30-year-old patients with skeletal Class Ⅰ or Ⅱ or Ⅲ malocclusion	Growth modulation, camouflage, or jaw surgery	Dentist	Extractions options in Class Ⅰ malocclusion; Growth modulation, camouflage and jaw surgery in Class Ⅱ and Ⅲ malocclusion	Not mentioned	Machine learning (7 kinds of algorithm) (top 10 parameters with the highest contribution)	33 cephalometric variables and nonradiographic inputs	Mandible body dimension	None
Hunter Lee (2022) [35]	Surgery-decision	196	Skeletal Class III patients	Surgery or nonsurgery treatment	Dentist	Nonsurgery group and surgery group	Not mentioned	Machine learning (random forest and logistic regression) (top 3 features with the highest importance scores in the specific algorithm)	60 cephalometric variables and nonradiographic inputs	L1-MP angle	Smaller
Ying-Chen Chen (2023) [54]	Surgery-decision	200	Adult aged over 20 years old with skeletal Class III malocclusion	Two-jaw surgery with the surgery-first approach (SFA) or orthodontic-first approach (OFA)	Based on the initial model manipulation and surgical occlusion management	The surgery-first approach group and orthodontic-first approach group	Not mentioned	Logistic regression analyses	2 cephalometric variables and noncephalometric inputs	L1-MP angle	Patients with a larger angle tend to be treated by OFA.
Jieni Zhang (2023) [55]	Practice guidance	198	Severe skeletal Class III patients (ANB ≤ −4°)	Surgery treatment	None	None	Not mentioned	ANOVA	13 cephalometric variables	The angle between the long axis of the mandibular symphysis and L1	None

RME, rapid maxillary expansion; FM, facemask; HG, headgear; CC, chin-cup; FUN, functional appliance; FIX, fixed appliance.

**Table 3:** Studies evaluating the association of cranial base and soft tissue with radiographic features on cephalograms using conventional statistical methods or machine learning approaches.

Author (Year)	Application	Sample Size	Inclusion	Treatment	Judgment Criteria	Type of Outcome	Period	Architecture	Input	Significant Output	Trend for Poorer Result/Extraction/Surgery Need (Cut-Off Point)
Cranial base											
Chi Bui (2006) [12]	Facial type	309	Patients with skeletal Class Ⅲ malocclusion	None	Cluster analysis	5 clusters representing distinct subphenotypes	Not mentioned	Cluster and principal component analyses	67 cephalometric variables	S-N, FH-SN angle	None
A. Stensland (1988) [11]	Growth prediction	91	4 to 9-year-old children with normal jaw relationship or skeletal Class Ⅲ malocclusion	Retractor + CC	Positive overjet	Success, relapse	5 to 18 months	Discriminant analysis	35 cephalometric variables	NSBa angle	Smaller
Matthew A. Ghiz (2005) [48]	Growth prediction	64	Children with skeletal Class Ⅲ malocclusion	RME + FM	A positive overjet and a Class Ⅰ molar relationship	Success, relapse	At least 3 years after treatment	Regression analysis	18 cephalometric variables	Co- GD line	Smaller
Angelika Stellzig-Eisenhauer (2002) [30]	Surgery-decision	175	Adults with skeletal Class Ⅲ malocclusion	Surgery or nonsurgery treatment	Dentist	Nonsurgery group and surgery group	Not mentioned	The discriminant function model	20 cephalometric variables	S-N	Smaller
Tiziano Baccetti (2004) [37]	Growth prediction	42	Children with skeletal Class Ⅲ malocclusion	RME + FM	The presence of Class III permanent molar relationship and negative overjet were defined as unsuccessful.	Success, relapse	6.5 years on average	Discriminant analysis	19 cephalometric variables and nonradiographic inputs	BaT–SBL angle	Larger
Janka Kochel (2011) [31]	Surgery-decision	69	Adults with skeletal Class Ⅲ malocclusion	Surgery or nonsurgery treatment	Dentist	Nonsurgery group and surgery group	Not mentioned	A discriminant analysis	19 cephalometric variables	NSAr angle	Smaller
Samim Taraji (2023) [8]	Surgery-decision	182	Adults with skeletal Class Ⅲ malocclusion	Surgery or nonsurgery treatment	Dentist	Nonsurgery group and surgery group	11 to 70 months	Machine learning (XG boost analysis) (with top 3 weights in XGBoost analysis	40 cephalographic variables and nonradiographic inputs	NBa-FH angle	Larger
Soft tissue											
Chi Bui (2006) [12]	Facial type	309	Patients with skeletal Class Ⅲ malocclusion	None	Dentist	5 clusters representing distinct subphenotypes	Not mentioned	Cluster and principal component analyses	67 cephalometric variables	N’perp-UL; N’perp-LL, N’perp–Pog’	None
A-Bakr M. Rabie (2008) [56]	Surgery-decision	25	Around 17-year-old patients with skeletal Class Ⅲ malocclusion	Surgery or nonsurgery treatment	Dentist	Nonsurgery group and surgery group	Not mentioned	Discriminant analysis	28 cephalometric variables	Holdaway angle	Smaller (12 degrees)
Hicham Benyahia (2011) [57]	Surgery-decision	47	Adults with skeletal Class Ⅲ malocclusion	Surgery or nonsurgery treatment	Dentist	Nonsurgery group and surgery group	Not mentioned	Stepwise discriminant analysis	27 cephalometric variables	Holdaway angle	Smaller (7.2 degrees)
Sara Eslami (2018) [33]	Surgery-decision	65	Adults with moderate skeletal Class Ⅲ malocclusion	Surgery or nonsurgery treatment	Dentist	Nonsurgery group and surgery group	Not mentioned	Stepwise discriminant analysis	24 cephalometric variables	Holdaway angle	Smaller (10.3 degrees)
Hunter Lee (2022) [35]	Surgery-decision	196	Skeletal Class III patients	Surgery or nonsurgery treatment	Dentist	Nonsurgery group and surgery group	Not mentioned	Machine learning (random forest and logistic regression) (top 3 features with the highest importance scores in the specific algorithm)	60 cephalometric variables and nonradiographic inputs	Holdaway angle	Smaller

RME, rapid maxillary expansion; FM, facemask; CC, chin-cup.

**Table 4:** Studies evaluating the radiographic features on CBCT and MRI.

Author (Year)	Sample Size	Inclusion	Architecture	Radiographic Feature
CBCT				
Chun-Yuan Huang (2016) [71]	96	18 to 45-year-old patients with normal dentation, retrognathism, and prognathism	ANOVA	The distance from the outer/buccal edge of the mandibular canal to the inner surface of the buccal cortex, and the distance from the lingula of the ramus to the dorsal root of the first molar
Ki-Jun Kim (2018) [72]	120	10 to 20-year-old patients with skeletal Class Ⅰ or Ⅱ or Ⅲ malocclusion	ANOVA	Cortical, cancellous, and total bone densities
F. Kalabalik (2020) [73]	30	Adult patients with Class Ⅰ or Class Ⅲ dentoskeletal patterns	*T*-test or Mann–Whitney U test	Thickness of the buccal cancellous bone, the distance from buccal aspect of mandibular canal (MC) to outer buccal cortical margin of mandible, the distance between superior aspect of MC and alveolar crest and the distances between first molar and the distal margin of mental foramen
Petra Santander (2020) [63]	111	Adult patients with skeletal Class Ⅰ or Ⅱ or Ⅲ malocclusion	MANOVA	Condylar dimension, antero–posterior, and medio–lateral inclination angles
MRI				
Serkan Görgülü (2011) [74]	66	Around 17-year-old patients with skeletal Class Ⅰ or Class Ⅲ malocclusion	ANOVA	Tongue posture and movement
W-S Jung (2013) [75]	460	Adult patients with skeletal Class Ⅰ or Ⅱ or Ⅲ malocclusion	ANOVA	TMJ disk position
Hatice Gökalp (2016) [76]	76	Around 10-year-old children with skeletal Class Ⅰ or Ⅱ or Ⅲ malocclusion	ANOVA	TMJ disk and condylar position
Daniella Torres Tagawa1 (2023) [77]	105	Children in CVS1&2 period with normal occlusion or skeletal Class Ⅲ malocclusion	ANOVA model or Kruskal–Wallis test or Cochran–Mantel–Haenszel test	TMJ articular disc position and shape

## Data Availability

Not applicable.

## Supplementary Materials

The following supporting information can be downloaded at: https://www.mdpi.com/article/10.3390/diagnostics14050544/s1, Table S1: Description of the critical landmark, plane, and measurement on radiograms.
