# Supplementary material for: Radiographic Imaging for the Diagnosis and Treatment of Patients with Skeletal Class III Malocclusion
Source: Diagnostics (Basel). 2024 Mar 4;14(5):544. doi: 10.3390/diagnostics14050544 (PMC10931164; doi:10.3390/diagnostics14050544)
Supplement: Supplementary file 1 [file diagnostics-14-00544-s001.zip › diagnostics-2862425-supplementary.pdf]

Appendix

Table S1. Description of the critical landmark, plane and measurement on radiograms

**Measurements**	**Description**
A	The deepest point on the curvature of the maxillary alveolar process
Anterior facial height (AFH)	The vertical distance between N and Me
Anterior nasal spine (ANS)	The extreme anterior point on the maxilla
Articulare (Ar)	The point of intersection of the dorsal contour of the condylar head and the contour of the external cranial base
ArH	The distance between Ar and SH plane
B	The deepest point on the curvature of the mandibular alveolar process
Basion (Ba)	The most inferior posterior point on the sagittal plane on the anterior border of the foramen magnum
Condylar axis (CondAx)	An axis passing through C and Co
Condylar center (C)	The center of the condyle
Condylion (Co)	The most posterior and superior point of the mandibular condyle
Condylion intersection (Coi)	The intersection of Co perpendicular line tangent to posterior border of the ramus
Facial plane (FP)	A plane passing through N and Pog
Frankfurt-Horizontal (FH) plane	A plane passing through Po and Or
Great divide (GD) line	A vertical line passing through S, perpendicular to SH plane
Gnathion (Gn)	The most downward and forward point on the mandibular symphysis
Gonion (Go)	The midpoint of the mandibular angle between the mandibular ramus and corpus
Gonion Intersection (Goi)	The point of intersection between the mandibular and ramus planes
GZN angle	The angle formed by the SN plane and mandibular ramus plane
Infradentale (Id)	The point of the alveolar contact with the lower central incisor
Lower facial height (LFH)	The distance between ANS and Me
Lower incisor apex (L1A)	The root apex of the most anterior mandibular central incisor
Lower incisor (L1)	The tip of the crown of the most anterior mandibular central incisor
L1H	The distance between L1 and SH plane
Lower lip (LL)	Vermillion border of the lower lip
Menton (Me)	The extreme inferior point of the mandibular symphysis
Mandibular plane (MP)	A plane passing through Gn and Me

Mandibular ramus (Rm) plane	The line tangential to the posterior border of the mandibular ramus
Nasion (N)	The extreme anterior point on the fronto-nasal suture
N perp	Perpendicular to FH plane and passing through N
Soft tissue nasion (N′)	Most posterior point on the soft tissue contour on the area of the frontonasal suture
N′ perp	Perpendicular to FH plane and passing through N′
Occlusal plane (OP)	An imaginary plane that extends from the incisal edges of the front teeth and passes through the tips of the cusps of the posterior teeth
Orbitale (Or)	The deepest point on the infraorbital margin
Palatal plane (PP)	A plane passing through PNS and ANS
PH	The distance between PNS and SH
Posterior nasal spine (PNS)	Posterior nasal spine: the extreme posterior point on the maxilla
Porion (Po)	The upper midpoint point on the external auditory meatus
Pogonion (Pog)	The extreme anterior point of the mandibular symphysis
Pogonion intersection (Pogi)	The intersection of Pog perpendicular line tangent to inferior border of the mandible
Pterygoid point (Pt)	The extreme superior point of the pterygopalatine fossa
Sella (S)	The midpoint of sella turcica
Soft tissue pogpnion (Pog′)	Most anterior point on the soft tissue contour on the mentum
Stable basicranial line (SBL)	A plane passing through point T and tangent to the cribriform plate of the ethmoid bone
Sella horizontal (SH) plane	A horizontal line through S 7 degrees below SN plane
SN plane	A plane passing through S and N
T	Uppermost point at the junction of the frontal wall of pituitary fossa and tuberculum sellae
Upper incisor (U1)	The tip of the crown of the most anterior maxillary central incisor
Upper incisor apex (U1A)	The root apex of the most anterior maxillary central incisor
Upper lip (UL)	Vermillion border of the upper lip
